# Database Constraints Applied to Metabolic Pathway Reconstruction Tools

**DOI:** 10.1155/2014/967294

**Published:** 2014-08-17

**Authors:** Jordi Vilaplana, Francesc Solsona, Ivan Teixido, Anabel Usié, Hiren Karathia, Rui Alves, Jordi Mateo

**Affiliations:** ^1^Computer Science Department & INSPIRES, University of Lleida, Jaume II 69, 25001 Lleida, Spain; ^2^CEBAL, IPBeja Campus, 7800 Beja, Portugal; ^3^Department of Basic Medical Sciences & IRBLleida, Edifici Recerca Biomedica I, Universitat de Lleida, Avenida Rovira Roure 80, 25198 Lleida, Spain

## Abstract

Our group developed two biological applications, *Biblio-MetReS* and *Homol-MetReS*, accessing the same database of organisms with annotated genes. *Biblio-MetReS* is a data-mining application that facilitates the reconstruction of molecular networks based on automated text-mining analysis of published scientific literature. *Homol-MetReS* allows functional (re)annotation of proteomes, to properly identify both the individual proteins involved in the process(es) of interest and their function. It also enables the sets of proteins involved in the process(es) in different organisms to be compared directly. The efficiency of these biological applications is directly related to the design of the shared database. We classified and analyzed the different kinds of access to the database. Based on this study, we tried to adjust and tune the configurable parameters of the database server to reach the best performance of the communication data link to/from the database system. Different database technologies were analyzed. We started the study with a public relational *SQL* database, *MySQL.* Then, the same database was implemented by a *MapReduce*-based database named *HBase.* The results indicated that the standard configuration of *MySQL* gives an acceptable performance for low or medium size databases. Nevertheless, tuning database parameters can greatly improve the performance and lead to very competitive runtimes.

## 1. Introduction

Our group developed two biological applications,* Biblio-MetReS* (http://metres.udl.cat/index.php/2-uncategorised/13-biblio-metres-paper-published) [1] and* Homol-MetReS* (http://homolmetres.udl.cat/prod/) [2], respectively, acronyms of Bibliomet- and Homolog-based metabolic network reconstruction server. These applications rely on an in-house relational database [2]. The database was built by matching the* KEGG* (http://www.genome.jp/kegg/) gene names to their* NCBI* (http://www.ncbi.nlm.nih.gov) names and synonyms. The database includes full gene names and synonyms tables for approximately 1,500 organisms with fully sequenced genomes. The database was implemented using* MySQL* (http://www.mysql.com/), one of the most popular open-source databases, mainly managed with* SQL* (structured query language), a standard programming language designed for managing data held in a relational database.

*Biblio-MetReS* is a user-friendly tool implemented in Java, which does on-the-fly analyses of the full text of scientific documents that are freely available on the Internet, and uses that analysis for the automated reconstruction of gene/protein networks. Such an on-the-fly approach leads to high runtimes, because the analysis of scientific documents is very resource consuming. To circumvent this high runtime,* preprocessing* of the documents [3–5] is typically used.* Preprocessing* of texts/documents consists of analyzing the documents offline, extracting the relevant information, and creating an adequate table to contain that information inside the database.

*Homol-MetReS* is a web application that permits simultaneous large-scale reannotation, functional integration, and automatic comparisons of metabolic networks on a multiple full genome scale. It automates comparisons that would otherwise be done almost manually, for example, using PathBlast [6], KEGG [7], or MetaCYC [8]. The functionalities in* Homol-MetReS* that have the highest needs for processing power and database access optimization are the comparison between fully sequenced genomes and the classification of large numbers of homologous proteins according to specific biological process categories. As in the case of* Biblio-MetReS*, database access is a bottleneck in the runtime of the server.

The system architecture (a data-center) hosting both applications presents a single access point for the computing needs of the users being served, like the ones described in [9, 10]. As stated in [11], most current data-center infrastructures consist of services that are offered by a web browser anywhere in the world. A service request sent by a user is transmitted to a server running a web service, which is associated with an SLA (service-level agreement). An SLA is a contract that a customer and a service provider have negotiated and agreed to. In such a contract, the customer only pays for the resources and services used, according to negotiated QoS (quality of service) requirements at a given price. Job runtime is perhaps the most important performance index in a cloud computing context [12], and it largely depends on the database system.

The requirement to perform compute-intensive analytics on (semi)structured bulk datasets has pushed SQL-like centralized databases to their limits [13]. This fact, along with the highly parallel nature of these tasks, has led to the development of horizontal scalable, distributed nonrelational data stores, called* NoSQL* (http://nosql-databases.org/) databases. Google's Bigtable [14], Amazon's Dynamo [15], Facebook's Cassandra [16], LinkedIn's Voldemort [17], and* HBase* are a representative sample of such systems.

In favor of scalability and high availability,* NoSQL* systems serve a dual purpose: they can efficiently store and index* NoSQL* sets arbitrarily while enabling a large number of concurrent user requests. Among other reasons,* NoSQL* systems may not be the optimal solution when data-centers have no need to deal with large datasets. However, in order to provide the overall system with a negotiated SLA, optimizing the current SQL database system may be a challenge. In [18], the authors dealt with the challenge of optimizing a* MySQL* database for novelty detection, which is the process of singling out novel information from a given set of text documents, in order to optimize the database tables for up to 10 million records. In [19], the authors also used database optimization and* SQL* tuning in order to achieve better performance levels for health monitoring systems. In this paper, in addition to these proposals we propose the application of* preprocessing* in order to guarantee a negotiated SLA in web-based systems with a* MySQL* biological database.* Preprocessing* of texts/documents consists of analyzing the documents offline, creating a database where the interaction information for each document is stored and can be quickly accessed [3–5]. Response time to client requests is shorter because it just depends on the elapsed time in accessing the database.

In the Results section of the present work, comparisons between optimized and nonoptimized* MySQL* databases were performed. We found that performing specific optimization of some database server parameters led to a significant performance increase without having to migrate the entire system to a different approach, like a* NoSQL* system. We show this by comparing the* MySQL* database with a* NoSQL* one, implemented with* MapReduce* (http://research.google.com/archive/mapreduce.html) and* HBase*.

## 2. Related Work and Motivation

There are important tools in the literature for the automatic identification of cooccurrence of genes/proteins from Internet to assist in the reconstruction of molecular circuits by inferring the gene/protein entities and their cooccurrence, such as Laitor [3], iHOP [4], String [5], and* Biblio-MetReS*, released in March 2011 [1] by our group.

Table 1 summarizes the main differences between these tools.* Biblio-MetReS* is the only tool able to search for gene cooccurrences in sentences, paragraphs, and the overall document. This allows* Biblio-MetReS* to better assess the interactions between a pair of genes. In addition to the cooccurrence statistic methods,* Biblio-MetReS* also provides the *P* value associated with cooccurrence, reducing the likelihood of false negatives or false positives when looking for genes/proteins in the texts/documents.

The main drawback of* Biblio-MetReS* is the elevated elapsed time processing the documents downloaded from Internet to extract gene/protein interactions. iHOP, String, and others (see Table 1 from [1] and references therein) are quicker in responding to client requests because they implement the* preprocessing* of information (documents) technique offline.* Bilblio-MetReS* also incorporates that, but all work is performed online. In this paper we deal with the database efficiency by tuning the* MySQL* parameters and incorporating the* preprocessing*.

**Figure 1:**
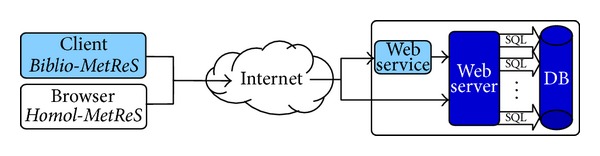
Cloud computing architecture.

Most current data-centers architecture consists of services that are offered and delivered through a service center that can be accessed from a web browser anywhere in the world (Figure 1).

In the* Biblio-MetReS* application, a desktop application acts as a client of the web service. In the case of* Homol-MetReS*, the client is the web browser itself. Requests delivered to the data-center are associated with an SLA. Our work is focused on guaranteeing the SLA (service-level agreement). On this occasion, the database performance was the main focus of our research. In doing so, we propose some solutions to provide a high level of QoS, thus determining a means to fix the SLA in accessing the database for the particular case of our subject applications.

The use of model organisms for research is a hallmark of scientific endeavour (e.g., [20]). The accumulation of fully sequenced genomes [21] and the advances in comparative genomics [22, 23] and computational systems biology [24] allow us to develop strategies that compare the protein or gene networks involved in the process of interest in order to establish similarities. These similarities can be used to predict, to a first approximation, the accuracy of extrapolating the behavior of specific processes between organisms. Testing this idea requires a thorough analysis of the molecular circuits in a well-known model organism and a comparison of these circuits to those in other living beings. In fact, a gap exists in systematically establishing how close different organisms are with respect to a given process, before choosing one of them as a model for studying that process [2]. Provided the* Biblio-MetReS* database of fully sequenced genomes,* Homol-MetReS* implements innovative functionality in such research field. Thus, as in the case of* Biblio-MetReS*, we are interested in providing* Homol-MetReS* with SLA guarantees.

## 3. *Biblio-MetReS*

**Figure 2:**
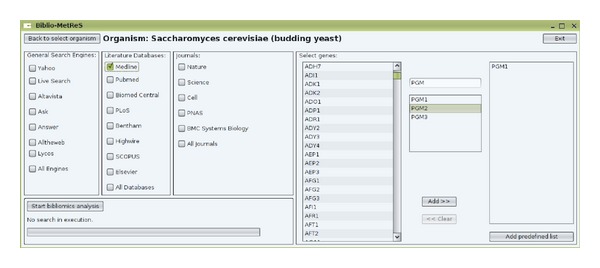
*Bilio-MetReS*. Main window.

*Biblio-MetReS* is a Java client-server application which needs a database in order to manage biological data efficiently, and more specifically information about organisms, genes/proteins, the processes in which the genes are involved, and the relationships between all these entities.* Biblio-MetReS* searches Internet for documents with data-mining techniques and operates as follows. Users must register to login in to* Biblio-MetReS*. After login, users must choose an organism to work with. The application loads all the genes in the database for the selected organism. Once this is loaded, the user is presented with the main window (Figure 2), where s/he can select data sources as well as genes to search for in those data sources across Internet. The data sources are as follows: General Search Engines (*Yahoo* (http://yahoo.com/),* Live Search* (http://www.bing.com/),* Ask* (http://www.ask.com/),* Answers* (http://www.answers.com/topic/statistics),* Altheweb* (http://altheweb.com/), and* Lycos* (http://www.lycos.com/)), literature databases (*Medline* (http://www.nlm.nih.gov),* PubMed* (http://www.ncbi.nlm.nih.gov/pubmed),* Biomed Central* (http://www.biomedcentral.com/),* PLoS* (http://www.plos.org/),* Bentham* (http://www.benthampapers.ucl.ac.uk/),* Highwire* (http://highwire.stanford.edu/),* SCOPUS* (http://www.scopus.com/), and* Elsevier* (http://www.elsevier.com/)), and journals (*Nature* (http://www.nature.com/),* Science* (http://www.sciencemag.org/),* Cell* (http://www.cell.com/),* PNAS* (http://www.pnas.org/), and* BMC Systems Biology* (http://www.biomedcentral.com/bmcsystbiol)).

Once the choices are made and the search has started, the tool identifies and downloads the documents from the selected data sources that contain the gene names used in the search query. Documents may be in HTML, PDF, or ASCII formats. Each document is then parsed to identify all other genes and gene synonyms (that were retrieved from the database) from the organism of interest that are also mentioned in the documents. A similar workflow is used to analyze biological processes in the documents. Entity cooccurrence patterns are analyzed and represented in several ways. For example, cooccurrence patterns can be represented using a 2D graph of the gene cooccurrence. The whole analysis process is done using a mixed strategy that combines newly found documents on the fly with preprocessed information retrieved from the database if a document has been found in previous searches. Basically, the performance of the tool depends on its effectiveness in processing documents and the online access to the database. Reference [1] provides the details on how cooccurrence is analyzed in documents.

## 4. *Homol-MetReS*

Proper functional identification of genes on a full genome scale for all organisms with fully sequenced genomes is only possible by transferring the functional information that is available for proteins from other organisms with fully sequenced genomes.* Homol-MetReS* is a web application that permits the functional information of a model organism to be extrapolated to other organisms where that process or circuit is hard to study or there is insufficient information.

*Homol-MetReS* provides three main functionalities. First, users can (re)annotate the function of each of the proteins in the proteome of an organism of interest with respect to many different classifications of biological functions. Although interesting, this functionality is independent of database performance, as the process is done via the analysis of preexisting text files and the creation of new ones to store transferred information.

**Figure 3:**
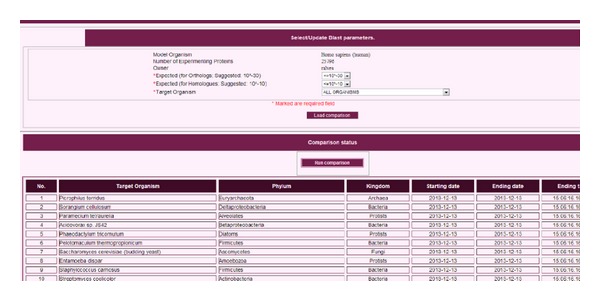
*Homol-MetReS*.

Second, users can automatically compare the sequences of the individual proteins from their proteome of interest to those of the full proteome from more than 1,200 other organisms that have fully sequenced and annotated genomes. Functional information from one organism can be transferred to another by the user, based on sequence homology. The process for doing so is illustrated in Figure 3. One of the bottlenecks in the computational process underlying this functionality is access to the database. This is so because many requests must be sent (in parallel when possible) to the database in order to compare the full proteome of an arbitrary number of organisms. These comparisons between organisms must be done within a reasonable time. We deal with the operations related to this kind of comparisons in the Results section. The database operations used in this functionality can mainly be classified as follows: (a) operations retrieving sequence information for the complete proteomes of any set of organisms contained in the database, (b) operations comparing information data of multiple organisms, and (c) operations to create temporary tables that contain information about the comparative analysis of the proteins between the organisms being analyzed. Third, the tables created in (c) are then used to represent heat maps that allow users to visually compare the similarity between the sets of proteins involved in specific biological processes in all the organisms being analyzed. Neither database access nor I/O is limiting for this functionality. Thus, its performance analysis was discarded in the Results section (Section 6).

## 5. Database Optimization

In order to optimize the interaction between* Biblio-* and* Homol-MetReS* and the database server, we designed a cloud architecture made up of 3 virtual machines. The first two contain the* Biblio-* and* Homol*-*MetReS* servers, respectively. The third virtual machine contains the database server. These three virtual machines were implemented using the* OpenStack* (http://www.openstack.org/software) framework. OpenStack is able to create as many virtual machines as needed in a dynamic manner. However, the main reason for choosing OpenStack was its freeware availability.

We now present the shared database used by* Biblio-MetReS* and* Homol-MetReS*, highlighting the most important tables. Then, an analysis of the improvements and optimization done on the database parameters is performed.

**Figure 4:**
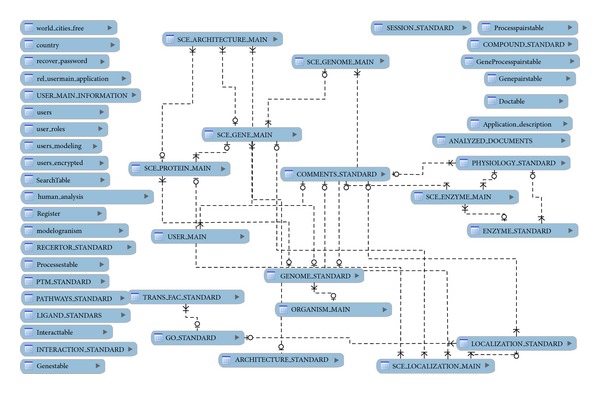
Simplified database schema.

Figure 4 shows a basic schema of the current relational database, implemented by means of* MySQL*.

According to the relational database definition, our database consists of a collection of tables organized according to the relational model with defined relationships with each other. There are the tables corresponding to organisms, genes, processes, and documents. These tables contain the information most frequently accessed by the applications and, therefore, the ones we focus on. Note that, in Figure 4, the tables corresponding to each of the different organisms present in the database are not explicitly listed. This is because there is one table for each defined organism and there are currently more than 1,200 different organisms in the database. In addition, although genes are organism-specific, biological processes are general and apply to all organisms. The relationship between genes and biological processes is also stored in organism-specific tables.

Also, there are other secondary tables in Figure 4 that are defined in order to support the applications. These tables include, for example, such user information as the* country* and* login name*. These tables are not considered, as they lack interest for our current purposes.

Table 2 shows an example of one of the tables that contains information about the proteins coded for in the genome of a fully sequenced organism contained in the central* MySQL* database. All such tables follow the same field structure, as shown in Table 2, where* XXX_ORG_ID* corresponds to the prefix of the organism.

Table 3 shows the description of the process table (*Processestable*) identified by* Biblio-MetReS* during the analysis of scientific documents. This table is general and stores information about biological processes associated with genes from one of the organisms with fully sequenced genomes currently included in the central database (e.g.,* Saccharomyces cerevisiae*,* Homo sapiens*,* Escherichia coli K12 MG1655*, or* Drosophila melanogaster*). The* XXX_isoform_id* column identifies which isoform of the gene is considered if the gene codes for more than one such isoform. The* status* column indicates whether the isoform has been experimentally confirmed or only predicted through bioinformatics analysis.

Table 4 summarizes the genes table (*Genestable*), which stores information regarding the genes that are found by* Biblio-MetReS* during the analysis of scientific documents. The* processes*,* typeofprocesses,* and* organism* columns connect the information found in the literature to the relevant organism and process contained in the database, while the other columns identify the document in which the process was identified.

Table 5 shows the description of the documents table (*Doctable*), which stores statistical information about the documents found by* Biblio-MetReS* in Internet in the data-mining search phase. The* Dockey* field is the unique ID of each document in the database. All other fields have information required to calculate mutual information for gene/protein cooccurrence in documents.

We now present the optimization we performed to the database. To facilitate understanding, the modifications related to* Biblio-* and* Homol-MetReS* are explained separately in the following sections.

### 5.1. *Biblio-MetReS* Optimization

Incoming documents found on the Internet are individually analyzed by* Biblio-MetReS*. Once a document has been analyzed, the results are stored in the database using the tables described above, among others. Subsequent searches that identify the same document will retrieve the preprocessed statistics stored in the database, thus avoiding repeating the time-consuming on-the-fly analysis. This process is known as* preprocessing*.

By implementing* preprocessing* we greatly decrease the time spent by the* Biblio-MetReS* application repeating the processing of documents. In contrast, much access to the database must be performed. On average, the database is accessed twice per new document to store statistical information for future use and 5 times per document to retrieve the statistical information from preprocessed documents when this information is required in subsequent searches.

Improvements in the database have little effect on the performance of* Biblio-MetReS*, as we will see in the Results section (Section 6). Because of this, we present and discuss the improvements made in database access in the following section,* Homol-MetReS* Optimization, where the gains are more significant.

### 5.2. * Homol-MetReS* Optimization

Several configuration parameters were adjusted to improve the database performance relating to the* Homol-MetReS* functionality. These parameters mainly affect the query caching done by the database server, hence improving query requests that will be executed more than once in a relatively short period of time.

These performance parameters were obtained using the* MySQLTuner* (https://github.com/major/MySQLTuner-perl) tool, which is a* Perl* (http://www.perl.org/) script that allows us to review a* MySQL* database and perform adjustments in order to increase performance and stability.

The main running parameters related to optimizing database performance can be obtained by executing the* MySQLTuner* benchmark, and they are as follows:* query_cache_size*,* table_open_cache*,* key_buffer_size*,* join_buffer_size*,* query_cache_limit,* and* innodb_buffer_pool_size*. The* query_cache_size* parameter determines the amount of memory allocated to caching query results. If set to* 0*, it disables the query cache. The optimal value depends on the system architecture. If set too high, it can provoke lock contention issues. The* table_open_cache* parameter determines the number of open tables for all threads. The number of file descriptors required by the* MySQL* server increases with* table_open_cache*. The* key_buffer_size* parameter determines the size of the buffer used for index blocks. The key buffer is also known as the key cache. The value of this variable indicates the amount of memory requested. Internally, the server allocates as much memory as possible up to this amount. It increases the value to obtain better index handling for all reads and multiple writes. The* join_buffer_size* parameter determines the size of each join buffer. The* query_cache_limit* parameter determines the maximum size of individual query results that can be cached.

## 6. Results

In this section, we present a set of results obtained from optimizing the application database. As data grows, there may be a drop in performance for access to the database shared by* Biblio-MetReS* and* Homol-MetReS*. That implies an increase in runtime and, therefore, a fall in QoS. In multiple situations, this entails noncompliance with the SLA agreement.

The current database server is located in a virtual machine, running on top of an HP Proliant DL165 G7 node with two Opteron 6,274 processors at 2.2 GHz with 16 cores each 192 GB of DDR3 RAM and 4.5 TB. The resources assigned to the virtual machine are 4 cores, 32 GB of RAM, and 525 GB of disk.

### 6.1. *Biblio-MetReS* Optimization

To measure the effect of implementing a* preprocessing* strategy we carried out a set of experiments where the execution runtimes of* Biblio-MetReS* with and without the strategy were measured.

**Figure 5:**
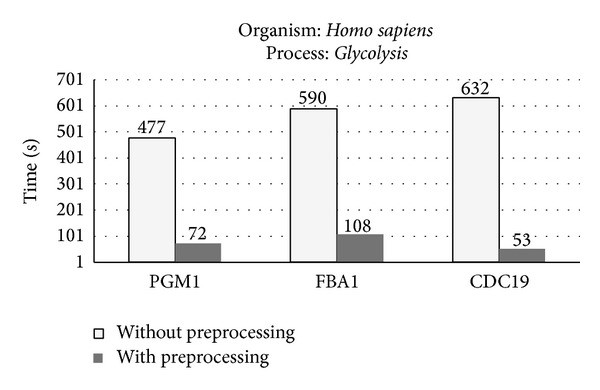
Runtimes with and without* preprocessing* for the* PGM1*,* FBA1,* and* CDC19* genes involved in the* Glycolysis* process of the* Homo sapiens* organism.

In the experiment, all the literature database sources were selected simultaneously for the search (see literature databases column in Figure 2).  Figure 5 only shows the results obtained for the* Homo sapiens* organism. The* PGM1*,* FBA1,* and* CDC19* genes involved in the* Glycolysis* process in* Homo sapiens* were used in the search. In the “*without preprocessing*” case, the runtime computed the search after the database had previously been emptied. During the procedure in doing the search, the database was updated with the statistical information of the documents found. Next, the same search was repeated (with the database containing almost all the documents). Thus, this operation shows the results “*with preprocessing*” activated. A large number of similar searches were done and the performance results were qualitatively similar (data not shown).

In general, performance gains, defined as the execution ratio between* preprocessing* and non*preprocessing*, ranged between 5 and 11, with an average improvement of 10. So, with* preprocessing* enabled,* Biblio-MetReS* was about 10 times faster than without* preprocessing*.

**Figure 6:**
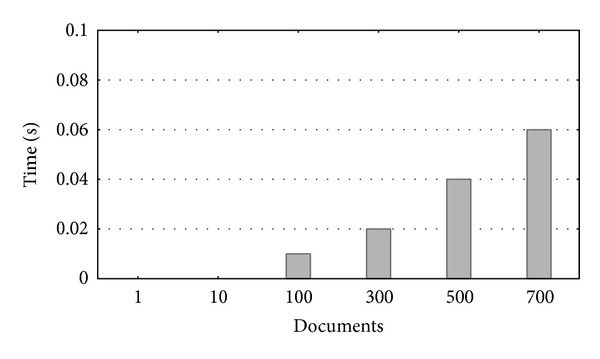
Runtime evolution when selecting from 1 to 700 simultaneous documents from the* Doctable* table.

Figure 6 shows the response time evolution when retrieving different numbers of documents from the* Doctable* database table (from 1 to 700 documents). It can be seen how response time increased slightly from 0 to 0.06 seconds when accessing larger numbers of documents. However, these variations were not very significant as runtime was always below 0.1 seconds, and therefore it had no real impact on overall performance. So we can affirm that* preprocessing* is a consistent improvement in the performance of* Biblio-MetReS* in the potential high-variability access to a data-center like the one where this tool was hosted.

### 6.2. Optimal Database Parameters

Several performance tests based on the current database usage were carried out in order to determine the optimal values for the database.  Table 6 shows a summary of the parameters thoroughly described in Section 5, containing their default values. Their optimal value (also in Table 6) was determined by using the* MySQLTuner* (https://github.com/major/MySQLTuner-perl) benchmark. Optimal values are referenced as “*Optimized*” and the default ones as “*Default.*”

We were interested in how runtime was affected by optimizing the values of the parameter set shown in Table 6 in the database server. In order to do the different optimization experiments, the current database server located in a virtual machine was replicated. Consequently, both database servers were located in their respective virtual machines, running on the HP Proliant DL165 G7 machine presented at the beginning of Results section.

Optimization of the parameters was done taking into account the database size, the maximum number of related tables in a search query in the database, and the length of tables and fields involved in it. These considerations led to the parameter limits shown in Table 6.

**Figure 7:**
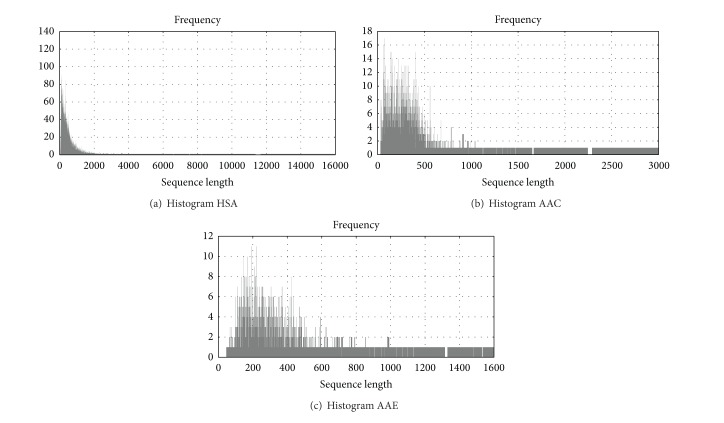
Histograms.

First, we determined the size of the database (6 GBytes), the main tables, and the fields to be used in the performance experiments. As a first optimization experiment we asked the biological question: “Is the distribution of protein sizes similar between organisms?” To answer this, we accessed each organism table and retrieved the size of the sequence of each annotated protein in its fully sequenced genome. The results are shown in Figure 7. We can see that the statistical distribution of protein sizes between organisms is qualitatively similar and has a long tail. This operation created a dataset that allowed us to compare and build histograms for the sizes of all proteins between two organisms, YPN and SPM. YPN is the acronym for* Yersinia pestis* Nepal516 (biovar Antiqua) and SPM stands for* Streptococcus pyogenes* MGAS8232 (serotype M18). The* ypn_ISOFORM_MAIN* and* spm_ISOFORM_MAIN* tables had 4,094 and 1,839 entries, respectively. However, the average protein size is organism-specific and increases rapidly with the number of proteins contained in the genome.

The* SQL* query used in this occasion was the following:

 SELECT OCTET_LENGTH (isoform_sequence) AS sequence,
   COUNT(∗) AS frequency
 FROM hsa_ISOFORM_MAIN
 GROUP BY OCTET_LENGTH (isoform_sequence);

This kind of operation entails database searches of type (a) from Section 4 (retrieving information of the different organisms).

**Figure 8:**
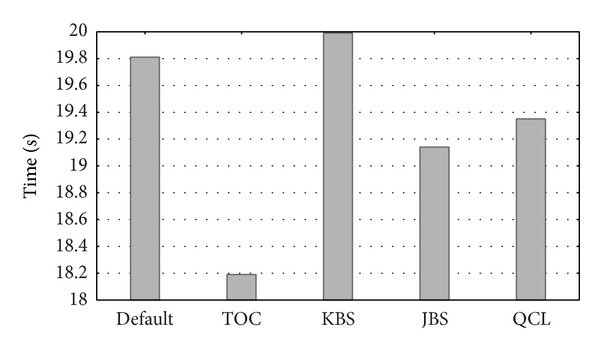
Runtimes for default and optimal values of the database server parameters. TOC: the* table_open_cache*, KBS:* key_buffer_size*, JBS:* join_buffer_size,* and QCL:* query_cache_limit*.

Experiments with the different parameter values of Table 6 were performed.  Figure 8 summarizes the results of optimizing the values of some of the parameters from Table 6. The* x*-axis represents different combinations of the parameters and the* y*-axis measures the runtimes (in seconds). In the first column, all the parameters were set to their default values. In the other columns, the enumerated parameters were set to their optimal value, and the remaining parameters were unchanged from their default values. This way the impact of each parameter on the performance could be independently measured. The sensitivity of runtime to changes in each parameter was, in increasing order of importance,* table_open_cache*,* join_buffer_size*,* query_cache_limit,* and* key_buffer_size*. Generally speaking, it can be said that the use of the optimal parameter values had a great impact on the overall system performance. The* query_cache_size* parameter is discussed separately below.

**Figure 9:**
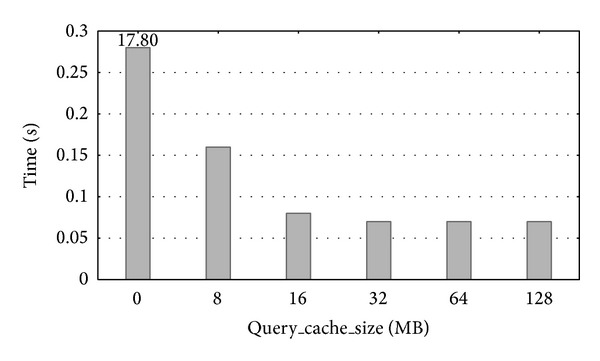
Runtime evolution when modifying the* query_cache_size* parameter.

We have also manually analyzed the effect of changing parameter values on the performance of the system to confirm the good behavior of the automated optimization done using* MySQLTuner*. In general, we observed that further increases in the parameter values led to no improvement in the performance of the applications. As an example, the effect of* query_cache_size* on the performance is shown in Figure 9. In this case, the evolution of the runtime by ranging* query_cache_size* between the default and the optimal values is represented. We see that increasing the query cache size beyond 64 MB had no effect on runtime, which justifies the choice of 64 MB as the optimal value by the* MySQLTuner* benchmark. However, this optimal value should account for database usage in order to avoid wasting resources. The* SQL* query used on this occasion was the following:

 SELECT COUNT(∗) FROM ypn_ISOFORM_MAIN
 LEFT JOIN (spm_ISOFORM_MAIN) ON (
   ypn_ISOFORM_MAIN.  isoform_sequence =
   spm_ISOFORM_MAIN.  isoform_sequence);

The type of this database operation is (b) from Section 4 (comparing information data of multiple organisms).

We conclude this section by remarking that the* SQL* queries took on average 0.1441 seconds using the default parameter values and 0.002 seconds using the optimized parameter values. This shows that our proposed optimization can lead to improvements in runtime of approximately three orders of magnitude.

### 6.3. Stressing the Database

**Figure 10:**
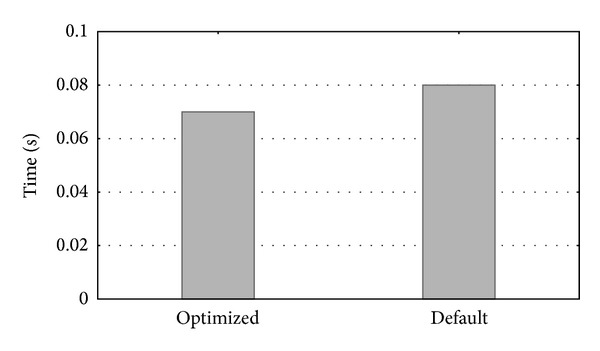
Runtime of a simple query.

Next, the optimization effect was measured by running a representative set of queries in* Bilio-* and* Homol-MetReS* using the default parameter values. We then ran the same set of queries using the optimized parameter values.  Figure 10 shows the runtime (in seconds) of the following* SQL* query used this time:

 SELECT ∗ FROM   Processestable;

The type of this database operation is (a) from Section 4 (retrieving information about the different organisms). This operation determines the number of individual biological processes defined in the database. Table* Processestable* had 10,933 entries. It is a very common query when selecting the process in the* Biblio-MetReS* application in the query definition, done before choosing the genes/proteins (see Figure 2).

Figure 10 shows that runtimes using the optimized parameter values were slightly faster than when using the default parameter. However, the performance improvement was small, and this could hardly be appreciated by the final user when operating the applications.

**Figure 11:**
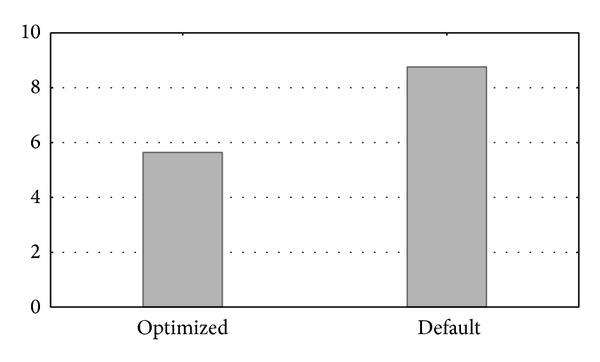
Runtime querying the* Homo sapiens* table.

Further experimentation was done with more complex queries to measure performance differences of both database's server configurations when more complex sets of operations are performed.  Figure 11 shows an example of such a search. Here, we can see a higher performance difference between the runtime of a query accessing a single table when using the optimized and the default parameter values.

In this case, the* SQL* query was the following:

 SELECT  isoform_sequence  FROM hsa_ISOFORM_MAIN;

The type of this database operation is (a) from Section 4 (retrieving information about the different organisms).

This experiment selected the sequences of all the proteins in the proteome of the* Homo sapiens* organism. The* hsa_ISOFORM_MAIN* table used had a total length of 25,796 entries. This is a common type of search in the database performed by users in both the* Biblio-* and* Homol-MetReS* applications. This operation is especially critical in the* Homol-MetReS* application, because similarities between genes are found by comparing the sequences retrieved from such a table with all the organisms. These comparisons give additional information or suggestions about organisms that lack this gene data. On this occasion, the gains of the optimized database compared to the nonoptimized one were quite significant (see Figure 11). We performed similar systematic searches in accessing other organism tables and the results were qualitatively similar to those shown in Figure 11 (data not shown).

**Figure 12:**
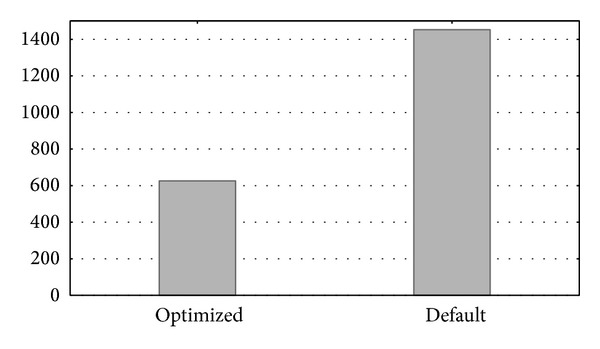
Runtime of a left join using 2 tables.

As stated above,* Homol-MetReS* compares sequences of protein between two organisms. To do so, the application needs to simultaneously access two tables. An example of the effect of parameter optimization on such operations is shown in Figure 12. This figure shows the runtime of a query that accesses two tables simultaneously. This operation creates a dataset that allows us to compare and build histograms for the sizes of all proteins between two organisms, ZMA and XLA. ZMA is the acronym for* Zea mays* (maize) and XLA stands for* Xenopus laevis* (African clawed frog).

The* SQL* query from Figure 12 is the following:

 SELECT zma_ISOFORM_MAIN.   isoform_sequence FROM zma_ISOFORM_MAIN
 LEFT JOIN (xla_ISOFORM_MAIN) ON (
 zma_ISOFORM_MAIN  .   isoform_sequence =
 xla_ISOFORM_MAIN  .  isoform_sequence);

The type of this database operation is (b) from Section 4 (comparing information data between multiple organisms).

The tables used in this experiment,* zma_ISOFORM_MAIN* and* xla_ISOFORM,_MAIN*, contain 17,821 and 10,681 entries, respectively. The* SQL* operation is a *LEFTJOIN*, which is very expensive computationally speaking. This experiment shows how, even in extreme stressed scenarios, the optimization of parameters leads to a strong performance improvement that decreases runtime by more than 50%. These results illustrate that our approach to optimizing database access parameters can lead to significant improvements in performance when running operations in* Homol-MetReS* and, to a lesser degree, in* Biblio-MetReS*.

### 6.4. Searching with* Preprocessing*

**Figure 13:**
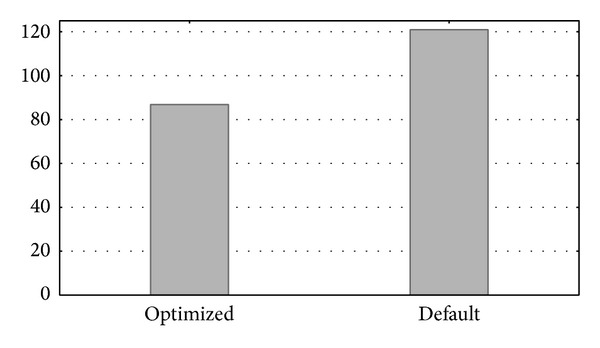
Runtime of a search query to the Processpairstable table.

Typically, the most time-consuming operations in* Biblio-MetReS* do not involve database access. Rather, they have to do with on-the-fly analysis of text in scientific documents. As stated above, improving the performance of the application for these operations was done by implementing a* preprocessing* strategy, as seen in Section 3. Nevertheless, as the number of preprocessed documents contained in the application's database increases, the effect of optimizing database access on the performance of the application will be increasingly significant. Therefore, it is important to estimate what the effect of optimizing database access might be in the future. To do so, we perform* Biblio-MetReS*-related searches using default versus optimized parameter values. The searches are done by selecting all defined biological processes from the table* Processpairstable*, which contains 33,731 entries.  Figure 13 shows that optimization of parameter values leads to significant improvements of about 30% in the runtime of this type of search.

The* SQL* query from Figure 13 is the following:

 SELECT ∗ FROM   Processpairstable;

The type of this database operation is (a) from Section 4 (retrieving information about the different organisms).

### 6.5. *MySQL* versus* MapReduce*

We were interested in quantifying the effect of using an optimized regular relational database implemented in* MySQL* versus the utilization of a* NoSQL* database, when the amount of available data is below the terabyte limit. To do so, we compared the performance of a set of searches run against our optimized database versus the same set of searches run against equivalent* NoSQL* implementation of our database. This implementation was done with* MapReduce* and* HBase*.* MapReduce* is a programming model for processing large datasets with a parallel, distributed algorithm on a supercomputing system (i.e., large cluster system).* HBase* is thought for random and realtime read/write access to large amount of data. The new database was mapped in another virtual machine with the same computational resources as the original database. Consequently, the two database servers were located in their respective virtual machines, running on the HP Proliant DL165 G7 machine presented at the beginning of Results section.

**Figure 14:**
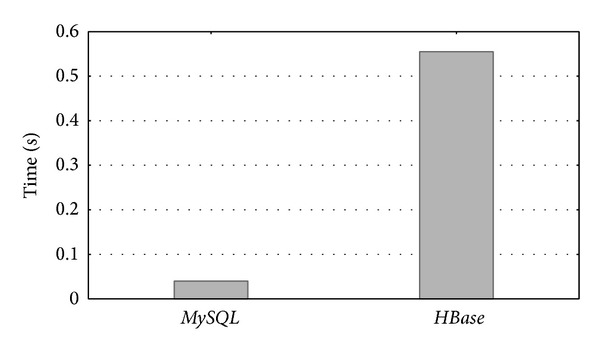
*MySQL* and* MapReduce* runtimes when accessing the* Processestable* table.

In the experiment, the* Processestable* table was accessed to compare the response times of a commonly used query on both systems.  Figure 14 shows the runtime (in seconds) of the following* SQL* query used this time:

 SELECT ∗ FROM   Processestable;

In summary,* MySQL* obtained the best performance.* MySQL* was one order of magnitude faster than* MapReduce/HBase* (averaged 60% faster). This justifies the selection of a typical database like* MySQL* as the technology for implementing the database server.

## 7. Discussion

The optimization experiments presented have been widely tested in order to determine the parameter values that maximize application performance. We present the main results and relate gains obtained with the kind of operations performed by* Biblio-MetReS* and* Homol-MetReS*. The optimal tunable values for the search in the database server were found empirically. The application whose performance was more extensively improved was* Homol-MetReS*.

A special search affecting the* preprocessing* was analyzed.* Preprocessing* reduced 10 times the* Biblio-MetReS* runtime. In addition, our proposed optimization can lead to improvements of three orders of magnitude. Even in extreme stressed scenarios, the optimization decreased runtime by more than 50%.

We also compared the performance of the database server (implemented as a relational* MySQL* database) against the same database implemented with the* MapReduce* in order to verify that the database was not large enough to apply* NoSQL* technologies.* MySQL* averaged 60% faster than* MapReduce/HBase*.

## 8. Conclusions

In this paper, we analyzed and optimized the performance of two biological cloud applications. We presented one main solution to achieve this improvement that consisted of analyzing and optimizing the database access parameters used by both applications in order to obtain a better level of performance. We successfully analyzed the current database demands and determined the parameters that could provide a greater positive impact on the system performance. The results show a significant improvement in the runtime of some of the most common and significant queries in a* MySQL* database, done by the biological applications presented,* Biblio-* and* Homol-MetReS*. The fully sequenced database used needs sizes of one or more orders of magnitude bigger than the one used by the biological applications used in this work to justify its use in* NoSQL* technologies. Preprocessing can further extend the use of* SQL* databases. Although this process was effective enough to obtain an acceptable performance, a* NoSQL* approach may be needed in the future as the database grows both in concurrent queries and size as more fully sequenced organism genomes are added.

## Figures and Tables

**Table 1:** Main differences between *Biblio-MetReS* (B), iHOP (i), String (S), and Laitor (L).

	B	i	S	L
Multiplatform	X	X	X	
Gene correlation in sentences	X	X		X
Gene correlation in paragraphs	X			
Preprocessing	X	X	X	
Statistical method: *P* value	X			
Statistical method: cooccurrence	X	X	X	X
List of synonyms	X	X	X	X
Interaction extraction between genes	X	X		X
Semantic interaction understanding		X		X
Graphical representation	X	X	X	

**Table 2:** Generic organism table description.

Field	Type	Null	Key	Default
XXX_isoform_id	char(20)	NO	PRI	
XXX_protein_id	char(20)	YES		NULL
XXX_gene_id	char(20)	YES		NULL
XXX_org_id	char(20)	YES		NULL
XXX_kegg_id	char(30)	YES		NULL
isoform_no	int(5)	YES	MUL	NULL
XXX_gene_symbol	varchar(50)	YES		NULL
XXX_prot_name	varchar(500)	YES		NULL
XXX_prot_synonym	varchar(500)	YES		NULL
ncbi_ref_id	char(25)	YES		NULL
gi_number	char(25)	YES		NULL
orf_start	int(5)	YES		NULL
orf_end	int(5)	YES		NULL
isoform_sequence	blob	YES		NULL
isoform_length	int(5)	YES		NULL
isoform_mol_wt	int(8)	YES		NULL
comments	char(200)	YES		NULL
status	char(50)	YES		NULL

**Table 3:** Generic process.

Field	Type	Null	Key	Default
Processes	varchar(100)	NO	PRI	NULL
*Dockey *	int(11)	NO	PRI	0
organism	varchar(100)	NO	PRI	NULL
individualOC	int(10)	NO		NULL
typeProcess	int(10)	NO	PRI	NULL

**Table 4:** Generic genes table description.

Field	Type	Null	Key	Default
Genes	varchar(100)	NO	PRI	
*Dockey *	int(11)	NO	PRI	0
organism	varchar(100)	NO	PRI	
individualOC	int(10)	NO		NULL

**Table 5:** Document.

Field	Type	Null	Key	Default
*Dockey *	int(11)	NO	PRI	NULL
title	text	YES		NULL
authors	text	NO		NULL
url	text	YES		NULL
doi	varchar(80)	YES		NULL
pmcid	varchar(80)	YES		NULL
pmid	varchar(80)	YES		NULL
benthamid	varchar(80)	YES		NULL
cellid	varchar(80)	YES		NULL
bmcid	varchar(80)	YES		NULL
nSentence	int(10)	NO		NULL
nParagraph	int(10)	NO		NULL

**Table 6:** *MySQL* optimization parameters.

Variable	Default value	Optimized value
query_cache_size	0 MB	64 MB
table_open_cache	64	4096
key_buffer_size	7996 MB	100 MB
join_buffer_size	128 KiB	1 MB
query_cache_limit	1 MB	16 MB
